# Suicidal jumper’s fracture – sacral fractures and spinopelvic instability: a case series

**DOI:** 10.1186/s13256-018-1668-1

**Published:** 2018-06-26

**Authors:** Daniela Nonne, A. Capone, F. Sanna, L. Busnelli, A. L. Russo, G. Marongiu, G. Dessì, A. Ferreli

**Affiliations:** 1 Department of Surgical Sciences, Orthopaedic and Traumatology Unit, University of Cagliari, Piazzale Ricchi n. 1, 09121 Cagliari, Sardinia Italy; 2 Orthopaedic and Traumatology Unit, Brotzu Hospital, Cagliari, Sardinia Italy

**Keywords:** Sacral fracture, Spinopelvic dissociation, Lumbopelvic fixation, Jumper’s fracture, Decompression

## Abstract

**Background:**

Sacral fractures with spinopelvic dissociation are rare, and hard to diagnose and treat. Fractures with a H- or U-shaped line are severely unstable, due to a dissociation of the spine and of the upper body of the sacrum from the pelvis. They are commonly due to high-energy trauma events, with severe neurological injuries in 80% of cases.

**Cases presentation:**

Five polytraumatized Caucasian patients, three women and two men (mean age: 34 years old) with spinopelvic dissociation were selected. All patients underwent level I–II examinations with radiographs and computed tomography total-body scans; all patients needed damage-control procedures. Sacral fractures were classified according to Denis and Roy-Camille classifications, and neurologic injuries of cauda equina according to Gibbons classification. Patients’ outcome was analyzed with the Majeed score. Definitive surgical treatment was appropriate for two patients (lumbar-pelvic fixation or transverse bar). Clinical and radiographic outcomes were analyzed periodically. Four patients survived, all of them suffered severe neurologic deficits. One case of osteomyelitis was treated with the removal of the fixation implants 23 months after the accident.

**Conclusions:**

Diagnosis of spinopelvic dissociation is frequently overlooked due to the severe associated injuries affecting these patients. In cases of a fall from high height, this lesion should be investigated with a lateral sacral radiographic view and computed tomography scan of the pelvis. If untreated, it can lead to severe and progressive neurologic deficit with muskuloskeletal deformities and persistent pain. Early decompression treatment is controversial, but an early lumbopelvic fixation is recommended. A correct diagnosis and early treatment can reduce morbidity and strongly improve the outcome of these patients.

## Background

Fracture-dislocation of the sacrum can be associated with a serious and highly unstable injury, the so-called “spinopelvic dissociation” or “suicidal jumper’s fracture” [1, 2]. This rare injury represents 3–5% of sacral fractures and is caused by high-energy accidents (falls from great height, road accidents with multidirectional dynamics) [3]. Frequently, the diagnosis is missed because of severe associated injuries (head trauma, thoracoabdominal trauma, kidney and bladder injuries) [2, 4].

Spinopelvic dissociation is a transverse fracture of the sacrum with a longitudinal transforaminal bilateral fracture, which causes a dislocation of the spine and the upper body of the sacrum from the pelvis and the sacral wings [2]. Therefore, the lumbar spine and the upper body of the sacrum can rotate and flex due to the psoas muscle and to gravity [5]. This anatomic-functional condition involves a sacral hyperkyphotic deformity [5], which is associated to severe neurologic injuries, such as cauda equina syndrome and radicular or plexus lesions [2].

In 1985, Roy-Camille, who first described this kind of fracture, called them “jumper’s fractures” and he divided them into three groups, based on the level of displacement on the sagittal plane [1, 4]. Subsequently, Strange-Vognsen and Lebech described another kind of fracture, characterized by a segmental comminution of the S1 vertebral body [1, 4].

Based on Denis’ classification, injuries of zone 2 and zone 3 are more frequent and there is an higher incidence of neurological lesions, such as anesthesia and sphincter deficit (97% of cases) [1, 6]. Among transverse fractures involving these areas, the morphologic patterns with a U-shaped and H-shaped line are typical [7].

Treatment of these fractures needs restoration and fixation of the connection between the lumbar spine and pelvis. The first step is the provisional fixation of the pelvis with angiographic embolization of arterial lesions, if necessary [8]. Today, nonsurgical treatment is considered obsolete. It consisted of a long-term supine immobilization without weight-bearing for 2 to 4 months, which could lead to general and local complications (such as pulmonary embolism, pneumonia, decubitus lesions) [8].

Early reduction and internal fracture fixation improves the healing process of neurologic defects and reduce the rate of comorbidities related to prolonged immobilization [8].

## Cases presentation

From 2009 to 2016, five polytraumatized Caucasian patients (M:F = 2:3), with a mean age of 34 years (range 25 to 49) were selected. Three of them had tried to commit suicide. They underwent radiographs of the pelvis (anteroposterior [AP], inlet, outlet, left lateral [LL] views), computed tomography (CT) total-body scans, angio-CT and damage-control procedures (potential external fixation and embolization of arterial lesions). All patients were comatose (Glasgow Coma Scale [GCS]: range 4–6), so early full neurologic examination was not achievable.

Sacral injuries were classified according to Denis and Roy-Camille classification (Table 1). Neurologic lesions of cauda equina were analyzed according to Gibbons classification (Table 2): all patients showed severe neurologic lesions: cauda equina syndrome (*n* = 3) and bilateral radicular L5–S1 deficit (*n* = 4).

**Table 1:** Data of the patients

Case	Denis	Roy-Camille	Morphology	Treatment
1	Zone 3	Type IV	H	EF in damage control (exitus)
2	Zone 3	Type III	H	Lumbar-iliac fixation (timing 17 days)
3	Zone 3	Type II	U	Definitive EF
4	Zone 3	Type III	U	Lumbar-iliac fixation and ilium-sacral (timing 9 days)
5	Zone 3	Type II	H	Lumbar-iliac fixation + EF (timing 12 days)

*EF* external fixation

**Table 2:** The Gibbons Classification of cauda equina impairment

Type	Neurological deficit	No. of patients
1	None	0
2	Paresthesias only	0
3	Lower extremity motor deficit	4
4	Bowel/bladder dysfunction	3

Functional outcomes were evaluated with the Majeed score [9] (Table 3). Mean follow-up was 20 months (range 12–36).

**Table 3:** Majeed Score

Case	Denis	Roy-Camille	Morphology	Treatment	Majeed Score	Follow-up (months)
1	Zone 3	Type IV	H	EF in damage-control (exitus)	–	–
2	Zone 3	Type III	H	Lumbar-iliac fixation (timing 17 days)	64	36
3	Zone 3	Type II	U	Definitive EF	100	18
4	Zone 3	Type III	U	Lumbar-iliac fixation and ilium-sacral (timing 9 days)	65	14
5	Zone 3	Type II	H	Lumbar-iliac fixation + EF (timing 12 days)	57	12

*EF* external fixation; *H* H-shaped; *U* U-shaped

**Fig. 1:**
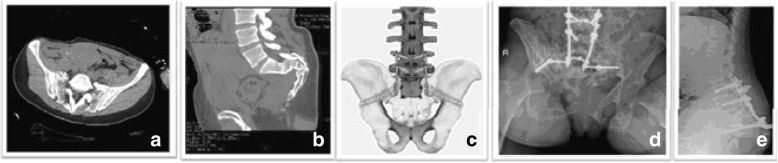
Case report: woman, 48 years. **a**-**b** H-shaped line fracture of the sacrum (Roy-Camille type III) and spinopelvic dissociation; (**c**) spinopelvic fixation [2]; **d**-**e** radiographic follow-up after 22 months

Every patient (*n* = 5) had a zone 3 fracture by Denis classification and, except for one case, the fracture was irreducible, complex, and displaced. One patient died 2 days after the accident due to pulmonary embolism. Posterior lumbar-iliac fixation was performed as definitive surgical treatment in two cases (Fig. 1). A case of external fixation was associated with a posterior lumbar-iliac fixation. One patient refused second-look surgery and he was treated only with external fixation.

The mean time interval between the accident and definitive treatment was 12 days (range 9–17), due to the severe clinical condition of the patients (acute kidney failure, severe respiratory failure, disseminated intravascular coagulation [DIC]). External fixation (EF) was removed after 45 days in order to proceed with posterior stabilization, or in the case of EF as definitive treatment, it was removed after 60 days.

Patients were rehabilitated early through passive mobilization and active assisted exercise of the lower limbs; they were then progressively discharged to an intensive neurorehabilitation center.

In the case of EF as definitive treatment, the patient achieved full weight-bearing in 4 months; partial recovery of sphincter function was shown in 12 months and full recovery in 18 months. The authors believe this excellent radiographic and functional result was due to the minimal displacement of the fracture which, with well-timed care, permitted healing, and neurologic recovery without complications.

Only one case (number 5 in Table 1) showed healing of the sphincter deficit in 6 months, but no recovery of the radicular L5–S1 deficit. The other two patients did not show any sign of healing of the neurologic deficit in the last follow-up. In these cases, indeed, complexity and displacement of fractures were so important that the authors presume a severe radicular lesion, which compromised neurologic healing.

With regard to complications, we had one case of osteomyelitis, which required hardware removal after 23 months and intravenous antibiotic therapy for 2 months, with full recovery. No cases of symptomatic protrusion of iliac screws or implant breakage were recorded.

## Discussion

Sacral fractures are overlooked in 30% of cases. Thus, this kind of injury should always be investigated in cases of falls from high height, with pelvic and sacral pain, lumbosacral hematoma (Morel-Lavelle lesion), sphincter deficit, or other neurologic lesions [8]. Pelvic and sacral radiographs in lateral view and CT scan are mandatory.

The severity of neurologic lesion depends on the complexity and displacement of the sacral fracture. Vaccaro *et al.* [8] stated that, if there was full bilateral neurologic damage or radicular avulsion, chances of healing were low.

Surgical recommendations and the timing of decompression are still questionable. The literature suggests that spinopelvic fixation represents the best surgical treatment of these lesions. In 1994, Kach and Trentz first proposed an open-surgery approach to reduce and stabilize the lumbar spine and pelvis with pedicle and ilium-sacrum screws (or posterior transilium plates), which are linked with bars or cross-linked [7, 10]. This technique allows equal distribution of forces to the acetabulum and lumbar spine, leaving out the fracture area and permits the early mobilization of the patient [10]. Pedicle screws are fixed in L5 and/or L4, other screws are placed in the iliac bone through the posterior superior iliac spine in parallel to the sacroiliac joint [10]. The correct placement of the iliac screws is evaluated through an intraoperative fluoroscopic Judet view of the pelvis [10].

The fixation of further fractures of the anterior pelvis, if necessary, can be performed subsequently. In the case of evidence of neurologic injury, the decompression can be performed indirectly through the reduction of the fracture or directly with a laminectomy or foraminectomy within 24–72 h from the accident, in order to decrease the risk of further nerve damage [7, 10].

Better results regarding neurologic healing can be achieved only if the treatment is performed within 24–72 h. However, in polytraumatized patients, the concurrent severe neurologic damage does not always allow performance of early surgery [8, 10]. Therefore, nerve decompression should be done both indirectly, with reduction of the fracture, and directly, through laminectomy or partial foraminectomy with lumboiliac fixation [8, 10].

Yi and Hak stated that late decompression could be hard, because of fibrous scar tissue, and it could worsen the neurologic deficit [10].

In the authors’ experience, there were no cases of early or late decompression surgery, because patients underwent surgical treatment after 10 days, so this was contraindicated.

Schildhauer underlined that full recovery of a radicular deficit or of cauda equina was possible only if there was continuity of nerve roots or, in the case of partial deficit [7], just as the case of the patient who was treated with external fixation (Table 3), because of minimal displacement of the fracture.

Moreover, Schildhauer *et al.* [2], and Yi and Hak [10] demonstrated an improvement of neurologic deficit in 80% of surgically treated patients independent of the kind of treatment. In 86% of patients with radicular compression, a partial recovery of sphincter function was appreciated after surgery with decompression and lumboiliac fixation; in the case of complete lesion or radicular avulsion only 36% of patients showed partial healing [10].

Schildhauer *et al.* reported deep infection in 16% of patients, which developed into chronic osteomyelitis in 47% [2].

Symptomatic breakage of implants without loss of reduction and stability was reported in 33% of cases [2].

## Conclusions

Early diagnosis and treatment of spinopelvic dissociation are crucial for a proper management of severe associated lesions and they can lead to a better recovery of neurologic deficit and better quality of life.

During the diagnostic process, when there is suspicion of this injury, proper radiographic assessment and CT scans are mandatory.

Posterior spinopelvic surgical stabilization, with or without decompression and fixation of anterior pelvis, represents the “gold standard” for this type of injury: this technique guarantees stability, neutralizes flexion deformation, allowing early mobilization of the patient, ensuring a reduction of comorbidities and a better recovery of neurologic lesions [2, 3].
